# Ca^2+^-Crosslinked Alginate Network Attenuates Starch Digestibility and Postprandial Glycemic Response in Rice Starch Gels

**DOI:** 10.3390/foods15122146

**Published:** 2026-06-14

**Authors:** Jie Tian, Nan Wang, Chen Song, Fanhua Kong, Chengrong Wen, Zedong Jiang, Shuang Song

**Affiliations:** 1 SKL of Marine Food Processing & Safety Control, National Engineering Research Center of Seafood, Collaborative Innovation Center of Seafood Deep Processing, National & Local Joint Engineering Laboratory for Marine Bioactive Polysaccharide Development and Application, Liaoning Key Laboratory of Food Nutrition and Health, School of Food Science and Technology, Dalian Polytechnic University, Dalian 116034, China; 2 College of Ocean Food and Biological Engineering, Jimei University, Xiamen 361021, China

**Keywords:** rice starch, Ca^2+^-crosslinked alginate, gel structure, water distribution, starch digestibility, digestion kinetics, postprandial glycemic response, gastrointestinal transit

## Abstract

Rice starch (RS) is widely consumed, but is usually rapidly digested, which may increase postprandial blood glucose levels. Therefore, regulating RS digestibility is important for development functional starch-based foods. In this study, sodium alginate (NaAlg) was incorporated into RS gels and subsequently crosslinked with Ca^2+^ to form a calcium alginate (CaAlg) network, and its effects on the physicochemical properties, digestion behavior, and physiological responses of RS gels were evaluated. Rheological measurement showed that the Ca^2+^-crosslinked alginate network increased the viscosity and viscoelastic moduli of RS gels. Low-field nuclear magnetic resonance analysis showed that the Ca^2+^-crosslinked alginate network reduced free water mobility. Structural characterization using Fourier-transform infrared spectroscopy, X-ray diffraction, and cold-field scanning electron microscopy shows that the Ca^2+^-crosslinked alginate network was associated with enhanced intermolecular interactions and a more continuous gel network, while all gelatinized samples exhibited predominantly amorphous structures. In vitro digestion experiments showed that the hydrolysis degree at 120 min decreased from 92.3% in RS to 85.6% in HCaAlg/RS. The rapidly digestible starch content significantly decreased from 72.4% to 68.4% (*p* < 0.05), while resistant starch significantly increased from 7.7% to 14.4% (*p* < 0.05). First-order kinetic fitting showed that C_∞_ significantly decreased from 93.0% to 86.0%, and k significantly decreased from 0.027 to 0.013 min^−1^ (*p* < 0.05). In vivo experiments showed that the Ca^2+^-crosslinked alginate/RS gels were associated with a lower postprandial glycemic response, with the incremental area under the curve significantly decreased from 747.2 to 591.7 mmol·min/L (*p* < 0.05), and the intestinal propulsion rate decreased from 89.6% to 75.3% (*p* < 0.05). These results suggest that Ca^2+^-crosslinked alginate network formation may modulate the structural properties, digestion behavior, and digestion-related physiological responses of RS gels, providing a basis for the development of starch-based functional foods with improved glycemic control.

## 1. Introduction

Rice starch (RS) is widely consumed because of its excellent functional properties, such as thickening, gel formation, and texture improvement, and it is a major source of carbohydrates in the human diet [1]. However, RS is usually rapidly digested, resulting in a rapid release of glucose and a sharp increase in postprandial blood glucose levels [2]. This response is associated with metabolic disorders and reduced insulin sensitivity [3,4]. Therefore, regulating the digestibility of RS has become an important strategy for developing functional foods with improved glycemic control.

The digestibility of starch is closely related to its structural characteristics and physicochemical properties [5]. During gelatinization, the native crystalline structure of starch is disrupted, forming a predominantly amorphous network that is more susceptible to enzymatic hydrolysis [6]. In addition, the distribution and mobility of water within the gel system play crucial roles in molecular mobility, enzyme accessibility, and overall digestion behavior [7,8]. Therefore, modifying the structure and hydration environment of starch gels is considered an effective approach to regulate starch digestion kinetics.

Hydrophilic colloids have been widely used to regulate the functionality of starch by modifying gel structure and water behavior [9,10]. Alginate is a representative anionic polysaccharide that can be crosslinked with Ca^2+^ to form a CaAlg network [11,12]. This network structure may limit the mobility of starch chains and water molecules, thereby affecting mass transfer during digestion [13,14]. Previous studies have shown that alginate-based systems can reduce starch digestibility and attenuate postprandial blood glucose response [14,15,16]. However, most available studies have focused on alginate-encapsulated rice beads or starch–hydrocolloid interactions, while the relationships between the structural characteristics of Ca^2+^-crosslinked alginate/RS gel systems and their in vitro digestibility, postprandial glycemic response, and gastrointestinal transit remain insufficiently understood.

In this study, NaAlg was incorporated into an RS gel system and subsequently crosslinked with Ca^2+^ to form a CaAlg network, and the associations between this network formation and the physicochemical properties, in vitro digestibility, and in vivo physiological responses of RS gels were systematically investigated. Rheological analysis is used to evaluate the gel properties, and low-field nuclear magnetic resonance (LF-NMR) is employed to characterize water distribution and mobility. Fourier-transform infrared (FT-IR) spectroscopy, X-ray diffraction (XRD), and cryo-scanning electron microscopy (cryo-SEM) are used to analyze structural changes. In addition, in vitro digestion experiments are conducted to evaluate starch hydrolysis behavior, and in vivo experiments were performed to assess postprandial glycemic response and gastrointestinal transit in mice. By integrating physicochemical characterization with in vitro and in vivo evaluations, this study aims to provide a more systematic understanding of how CaAlg network formation is associated with the functional behavior of RS gels.

## 2. Materials and Methods

### 2.1. Materials

Sodium alginate (NaAlg) was obtained from Qingdao Mingyue Seaweed Group Co., Ltd. (Qingdao, China) with a viscosity-average molecular weight of 3.81 × 10^7^ Da and a viscosity of 761 mPa·s. Calcium chloride (CaCl_2_, analytical grade) and sodium acetate buffer (3 M, pH 5.2) were purchased from Macklin (Shanghai, China). Rice starch (RS, ≥99%) and amyloglucosidase (100,000 U/g, from *Aspergillus niger*) were supplied by Shanghai Yuanye Bio-Technology Co., Ltd. (Shanghai, China). Thermostable α-amylase (≥135 KNU/g, from *Bacillus licheniformis*) and pancreatin (from porcine pancreas; protease ≥ 125 U/mg, amylase ≥ 125 U/mg, lipase ≥ 10 U/mg) were obtained from Aladdin Biochemical Technology Co., Ltd. (Shanghai, China). Enzyme activities were expressed according to the manufacturers’ specifications.

All other chemicals used were of analytical grade unless otherwise stated.

### 2.2. Preparation of CaAlg/RS System

NaAlg powder was first dispersed in deionized water and heated at 80 °C for 60 min to obtain a uniform solution. Subsequently, RS powder was added, and the mixture was maintained at 80 °C for 40 min with continuous stirring. CaCl_2_ solution was then slowly introduced while continuously stirring the sample, followed by continued heating at 80 °C for another 20 min. During this process, NaAlg was crosslinked by Ca^2+^ released from CaCl_2_, leading to the formation of a CaAlg network within the RS gel system. The resulting homogeneous sample was cooled and stored for 16 h to promote network structure development. The final total solid concentration of the composite systems was maintained at 9% (*w*/*v*). The LCaAlg/RS system contained 1.16% NaAlg, 0.13% CaCl_2_, and 7.71% RS (*w*/*v*), whereas the HCaAlg/RS system contained 1.35% NaAlg, 0.15% CaCl_2_, and 7.50% RS (*w*/*v*). These ratios were selected based on preliminary experiments, as they allowed the formation of stable and homogeneous gel systems while maintaining sufficient starch content for subsequent analyses. Additionally, a suitable amount of RS was dispersed in deionized water to prepare a 9% (*w*/*v*) RS solution, which was heated at 80 °C for 60 min until fully gelatinized. The solution was then cooled and stored for 16 h under the same conditions and named RS.

### 2.3. Rheological Measurements

The determination of rheological properties was based on the method reported by Tian et al. [17], with slight modifications. Rheological properties were determined using a DHR-2 rheometer (TA Instruments, New Castle, DE, USA) equipped with a serrated parallel-plate geometry (40 mm diameter, 1000 μm gap). Prior to testing, samples were equilibrated at room temperature for 10 min.

The linear viscoelastic region (LVR) was identified by a strain sweep test (0.01–100%) at 1 Hz and 25 °C. Frequency sweep measurements were subsequently conducted within the LVR over a frequency range of 0.1–10 Hz to obtain storage modulus (G′) and loss modulus (G″). Steady shear measurements were performed at shear rates ranging from 0.1 to 10 s^−1^ to evaluate apparent viscosity.

### 2.4. Water Distribution Measurements

Water distribution and mobility were analyzed using low-field nuclear magnetic resonance (LF-NMR; MesoMR23-060V-I, Suzhou Niumag Analytical Instrument Co., Ltd., Suzhou, China). Approximately 2 g of sample was transferred into an NMR tube and measured at 32 °C. Transverse relaxation time (T_2_) was obtained using the Carr–Purcell–Meiboom–Gill (CPMG) pulse sequence.

Key parameters were set as follows: echo number (NECH) = 5000, number of scans (NS) = 4, recycle delay (Tw) = 5000 ms, and echo time (TE) = 1.0 ms. The spectral width was 100 kHz, and the resonance frequency was 21 MHz.

### 2.5. Freeze–Thaw Stability Measurements

The samples for freeze–thaw stability analysis were prepared by the method of Muadklay et al. with some modifications [18]. Approximately 5 g of the sample was placed in centrifuge tubes, frozen at −30 °C for 24 h, and subsequently thawed at 30 °C for 2 h. After thawing, samples were centrifuged at 8000× *g* for 15 min. The supernatant was removed, and the remaining gel was weighed. The water separation rate was calculated based on the mass difference before and after centrifugation. The procedure was repeated for three freeze–thaw cycles.

The water separation rate (%) was calculated according to the following formulation:

$$Water\;separation(\%)=\frac{{W_{1}-W}_{2}}{W_{1}}\times 100$$

where W_1_ and W_2_ represent the weight of the sample before and after centrifugation, respectively.

### 2.6. FT-IR Analysis

Fourier-transform infrared (FTIR) spectra were recorded using an FTIR spectrometer (PerkinElmer, Waltham, MA, USA). The freeze-dried sample (2 mg) was mixed with KBr (100 mg) and pressed into a thin pellet. The spectra were collected over a wavenumber range of 400–4000 cm^−1^ at a resolution of 4 cm^−1^ at room temperature [17].

### 2.7. XRD Measurement

X-ray diffraction (XRD) patterns were obtained based on the method reported by Yu et al. [19], with slight modifications. Freeze-dried samples were equilibrated at room temperature under saturated relative humidity for 24 h prior to analysis. The diffraction patterns were recorded using an XRD (D8 Advance, Bruker AXS, Karlsruhe, Germany) operated at 40 kV and 40 mA. Data were collected over a 2θ range of 4–40° at a scanning rate of 0.1°/s.

### 2.8. Microstructure Observation

The microstructure of the gel samples was observed using a cryo-scanning electron microscopy (cryo-SEM) system (PP3010T, Quorum Technologies Ltd., Laughton, East Sussex, UK). The temperatures of the SEM cold trap, cryo stage, preparation stage, and cryo preparation chamber were maintained at −175 °C, −140 °C, −140 °C, and −175 °C, respectively. The gel samples were placed on specimen holders, rapidly frozen by immersion in liquid nitrogen, and then transferred to the cryo-preparation chamber. The frozen samples were fractured to expose the internal structure, followed by sublimation at −90 °C for 30 min. The samples were subsequently sputter-coated with gold at a current of 10 mA for 40 s. The microstructure was then examined using a scanning electron microscope (SU8010, Hitachi Corporation, Tokyo, Japan) at an accelerating voltage of 10 kV.

### 2.9. In Vitro Digestibility Measurements

In vitro starch digestibility was determined based on the method described by Englyst et al. [20,21], with slight modifications. Briefly, 0.2 g of sample was mixed with 15 mL of sodium acetate buffer (0.5 M). Subsequently, 10 mL of freshly prepared enzyme solution containing α-amylase (150 U/mL), amyloglucosidase (100 U/mL), and pancreatin (0.02 g/mL) was added. The mixture was incubated in a shaking water bath at 37 °C and 100 rpm for 120 min. Independent digestion systems were prepared for each sampling time point (0, 20, 40, 60, 80, 100, and 120 min). At each time point, 0.2 mL of the digestion mixture was withdrawn and immediately mixed with 0.8 mL of absolute ethanol to terminate enzymatic activity. The released reducing sugars were determined using the 3,5-dinitrosalicylic acid (DNS) method and expressed as glucose equivalents. The Hydrolysis rate of digestion (HRD) was calculated based on the glucose content using the following equation:

$$HRD\;(\%)=\frac{G_{t}\times 0.9}{TS}\times 100$$

where $G_{t}$ is the mass of released reducing sugars expressed as glucose equivalents at digestion time t, TS is the initial dry starch mass in the digestion system, and 0.9 is the conversion factor from glucose to starch.

Based on the released glucose equivalents at 20 and 120 min, rapidly digestible starch (RDS), slowly digestible starch (SDS), and resistant starch were calculated as follows:

$$RDS(\%)=\frac{G_{20}\times 0.9}{TS}\times 100$$

$$SDS(\%)=\frac{\left(G_{120}-G_{20}\right)\times 0.9}{TS}\times 100$$

Resistant starch(%)=100−RDS−SDS

where $G_{20}$ and $G_{120}$ represent the mass of released reducing sugars expressed as glucose equivalents after 20 and 120 min of digestion, respectively. The term “resistant starch” was written in full to avoid confusion with the abbreviation RS for rice starch.

To further evaluate starch digestion kinetics, the hydrolysis curves were fitted using a first-order kinetic model:

$$C_{t}=C_{\infty }\left(1-e^{-kt}\right)$$

where $C_{t}$ is the hydrolysis degree at digestion time t, $C_{\infty }$ is the estimated equilibrium hydrolysis degree, and k is the first-order digestion rate constant. The parameters $C_{\infty }$ and k were obtained by fitting the HRD values at different digestion times.

### 2.10. Animal Experiments

SPF-grade C57BL/6J mice, 45 females (aged 10 weeks, 25 g ± 2 g) were procured from Liaoning Changsheng Biotechnology Co., Ltd. (Benxi, China; Laboratory Animal Production License No. SCXK (Liao) 2020-0001). The mice were allowed a period of acclimatization to their environment for 7 days prior to the initiation of the experiment, during which they were provided with the standard maintenance diet for mice. Throughout the study, all mice were maintained under controlled conditions at a temperature of 23 °C ± 2 °C and humidity levels of 50% ± 5%, following a light/dark cycle of 12 h each. All animal procedures were conducted in accordance with the Guidelines for the Care and Use of Laboratory Animals established by Dalian Polytechnic University of Technology, and the experiments received approval from the Animal Ethics Committee of Dalian Polytechnic University of Technology (ID DLPU2025SP006, Ethical Approval date 15 October 2025).

After acclimatization, the mice were divided into two independent experiments. 15 mice were used for the postprandial glycemic response experiment, and 30 mice were used for the gastrointestinal transit experiment. Mice were randomly allocated to the experimental groups. Because the samples differed in appearance and viscosity, formal blinding was not applied during sample administration or outcome assessment. The overall animal experimental design is shown in Figure S1.

#### 2.10.1. Experimental Grouping of Postprandial Glycemic Response

After one week of adaptive feeding, 15 C57BL/6J mice were randomly divided into three groups (*n* = 5): RS, LCaAlg/RS, and HCaAlg/RS. After a 12 h fast, immediate blood glucose testing was performed. Specifically, mice in each group were administered 0.1 mL of the corresponding sample via oral gavage. Based on the gavage volume and formulation of each gel system, the approximate starch amounts administered were 9.00 mg per mouse for the RS group, 7.71 mg per mouse for the LCaAlg/RS group, and 7.50 mg per mouse for the HCaAlg/RS group. Because CaAlg was formed through Ca^2+^-induced crosslinking of NaAlg within the gel system, CaAlg was not administered as an isolated raw material. All groups received the same gavage volume, but the starch dose was not identical among the groups. Blood glucose levels were measured at 0, 15, 30, 60, 90, and 120 min using a glucometer (GA-3, Sinocare Inc., Changsha, China) [22].

The incremental area under the blood glucose curve from 0 to 120 min was calculated using the trapezoidal rule and expressed as mmol·min/L, according to the following equation:

$$AUC=\sum_{i=0}^{n-1}\frac{\left[\left(G_{i}-G_{0}\right)+\left(G_{i+1}-G_{0}\right)\right]}{2}\times \left(t_{i+1}-t_{i}\right)$$

where $G_{i}$ is the blood glucose concentration at time $t_{i}$, $G_{0}$ is the fasting blood glucose concentration at 0 min, and $t_{i}$ represents the sampling time point.

#### 2.10.2. Experimental Grouping of Gastrointestinal Transit

After one week of adaptive feeding, 30 C57BL/6J mice were randomly divided into six groups (*n* = 5): 0 min-RS, 0 min-LCaAlg/RS, 0 min-HCaAlg/RS, 30 min-RS, 30 min-LCaAlg/RS, and 30 min-HCaAlg/RS. After a 12 h fast, mice in each group were administered 0.1 mL of the corresponding sample containing a green ink marker by oral gavage. The green ink marker was used to visualize gastrointestinal transit and appeared as a blue-green signal in the gastrointestinal tract. The same formulation-based dosing strategy described above was used for the gastrointestinal transit experiment. For the 0 min groups, mice were sacrificed immediately after gavage, and the gastrointestinal tracts were harvested to show the initial distribution of the ink marker. For the 30 min groups, mice were sacrificed 30 min after gavage and subjected to immediate laparotomy. In the 30 min group, the distance traveled by the charcoal meal (from the pylorus to the leading edge) and the total length of the small intestine were measured to determine the intestinal transit [23]. The intestinal propulsion rate was calculated only for the 30 min groups using the following equation:

$$Intestinal\;Propulsion\;Rate\;(\%)\;\;\;\;\;\;\;\;\;\;\;\;\;\;\;\;\;\;\;=\frac{Distance\;of\;charcoal\;migration\;(cm)}{Total\;length\;of\;small\;intestine\;(cm)}\;\times \;100\%$$

### 2.11. Statistical Evaluations

Data are expressed as mean values with standard deviations. Statistical analyses were performed using IBM SPSS Statistics, version 22.0 (IBM Corp., Armonk, NY, USA). Differences among groups were evaluated by one-way analysis of variance (ANOVA), followed by Duncan’s multiple range test for pairwise comparisons. Statistical significance was defined at *p* < 0.05. Figures were generated using OriginPro, version 8.5 (OriginLab Corporation, Northampton, MA, USA).

## 3. Results and Discussion

### 3.1. Ca^2+^-Crosslinked Alginate Network Enhances the Rheological Properties of RS Gel Systems

**Figure 1:**
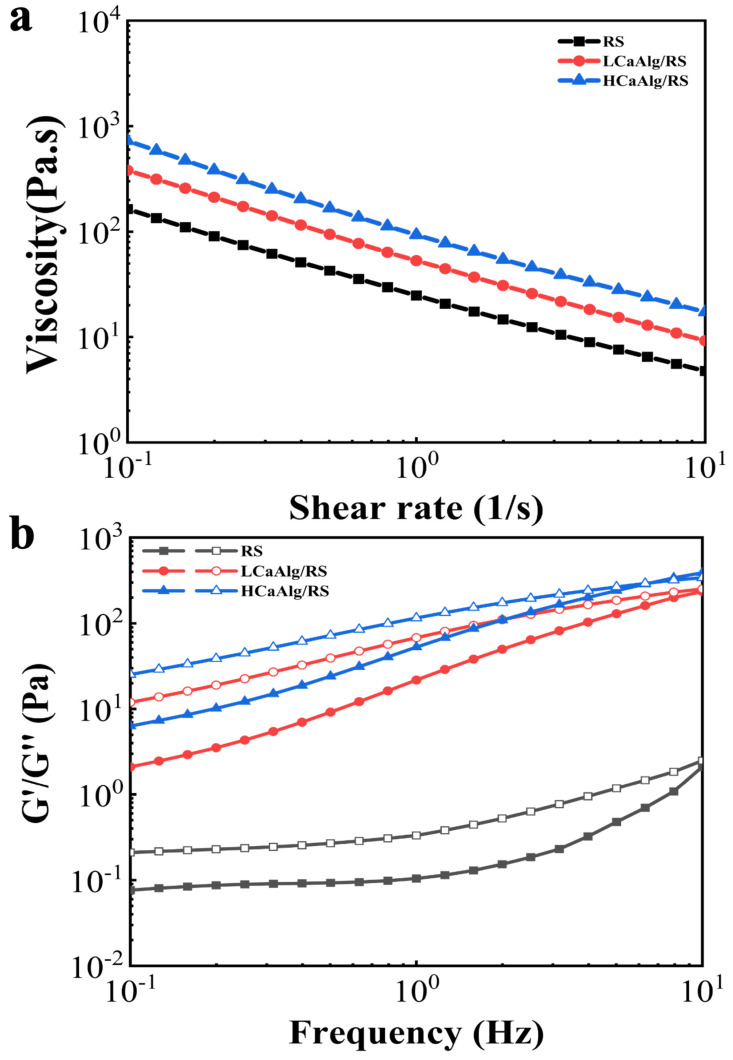
Rheological properties of the Ca^2+^-crosslinked alginate/RS gel system: viscosity as a function of shear rate (0.1−10 s^−1^) (**a**), and frequency dependence of storage modulus (G′) and loss modulus (G″) (**b**).

Rheological properties are widely used to characterize the physicochemical properties and structural behavior of starch gel systems [24]. Figure 1 shows the effect of Ca^2+^-crosslinked alginate network formation on the rheological behavior of RS gel systems. Figure 1a shows the relationship between apparent viscosity and shear rate. All samples exhibited shear-thinning behavior, with viscosity gradually decreasing as the shear rate increased. Compared with RS, LCaAlg/RS and HCaAlg/RS showed higher apparent viscosity, and HCaAlg/RS showed higher viscosity than LCaAlg/RS. Figure 1b shows the frequency sweep results at 25 °C. For all samples, the storage modulus (G′) and loss modulus (G″) increased with increasing frequency. Compared with RS, the Ca^2+^-crosslinked alginate–RS gel systems showed higher G′ and G″ values, and these values further increased in HCaAlg/RS. In addition, G′ remained higher than G″ over the entire frequency range.

The increase in viscosity and viscoelastic moduli indicates that Ca^2+^-crosslinked alginate network formation improved the structural integrity of the RS gel system. This effect may be related to Ca^2+^-mediated alginate crosslinking and non-covalent interactions between alginate and starch chains, which may contribute to a more stable gel framework and limit the mobility of starch chains. Previous studies have shown that sodium alginate can affect the gelatinization, rheological, and retrogradation properties of RS, indicating that alginate–starch interactions play an important role in determining the behavior of rice starch gels [25]. In the present study, the further crosslinking of alginate by Ca^2+^ may have contributed to the formation of a stronger gel network, as reflected by the increased viscosity and viscoelastic moduli of LCaAlg/RS and HCaAlg/RS. Similar enhancements in rheological properties have been reported in starch–hydrocolloid systems, where the incorporation of polysaccharides strengthens the gel structure by reinforcing network formation [26,27]. Overall, these results indicate that the Ca^2+^-crosslinked alginate–RS systems exhibited stronger rheological properties, providing a structural basis for its effects on digestion and physiological responses.

### 3.2. Ca^2+^-Crosslinked Alginate Network Modulates Water Behavior in RS Gel Systems

**Figure 2:**
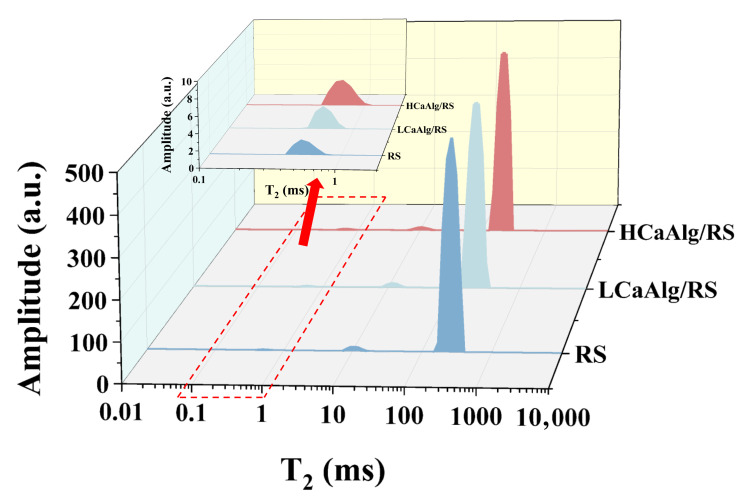
CPMG relaxation time of water distribution in Ca^2+^-crosslinked alginate/RS gel systems.

Water behavior is closely related to the network structure and stability of starch-based gel systems. LF-NMR provides a rapid and non-destructive method to evaluate water distribution and mobility. According to previous LF-NMR studies, a shorter transverse relaxation time (T_2_) is generally associated with lower water mobility and stronger restriction of water molecules within polymer matrices [28]. The water separation rate after freeze–thaw treatment was used as a supplementary macroscopic indicator of water retention capacity and is presented in Supplementary Table S1 [29,30]. As shown in Figure 2 and Table 1, the LF-NMR relaxation profiles of RS systems with different formulations are presented. The relaxation signals were assigned to bound water T_21_ (0.1–1 ms), non-migrating water T_22_ (1–100 ms), and free water T_23_ (>100 ms), and the low-relaxation-time region is shown in the enlarged inset of Figure 2. Among these components, T_23_ accounted for the largest proportion of the total signal, with A_23_ representing more than 95% of the total peak area. Therefore, T_23_ and A_23_ were used as the main indicators of free water mobility in the gel systems. Compared with RS, T_23_ decreased from 265.6 ms to 231.0 ms in both LCaAlg/RS and HCaAlg/RS. Meanwhile, A_23_ decreased from 1934.8 in RS to 1839.0 in LCaAlg/RS and 1770.9 in HCaAlg/RS. These results indicate that the Ca^2+^-crosslinked alginate/RS gel systems contained a lower proportion of mobile free water and showed restricted water mobility within the gel matrix.

In addition, the freeze–thaw stability results, which are presented in Supplementary Table S1, further supported the water retention behavior of the gel systems. The water separation rate decreased from 40.3% in RS to 38.4% in LCaAlg/RS and 35.3% in HCaAlg/RS, indicating that the Ca^2+^-crosslinked alginate–RS gel systems exhibited improved water retention capacity.

The reduction in the A_23_ indicates that Ca^2+^-crosslinked alginate network formation reduced the proportion of free water and promoted water retention within the gel matrix. This effect may be attributed to the interaction between alginate and starch chains, which enhances water binding and limits the mobility of water molecules. Similar behavior has been reported in starch–hydrocolloid systems, where polysaccharides enhance water immobilization through intermolecular interactions [31,32]. The supplementary freeze–thaw stability results further supported this trend, showing improved water retention capacity in the Ca^2+^-crosslinked alginate/RS gel systems (Table S1). Overall, these results indicate that Ca^2+^-crosslinked alginate network formation contributed to water stabilization within the RS gel matrix.

### 3.3. Ca^2+^-Crosslinked Alginate Network Influences the Structural Characteristics of RS Gel Systems

**Figure 3:**
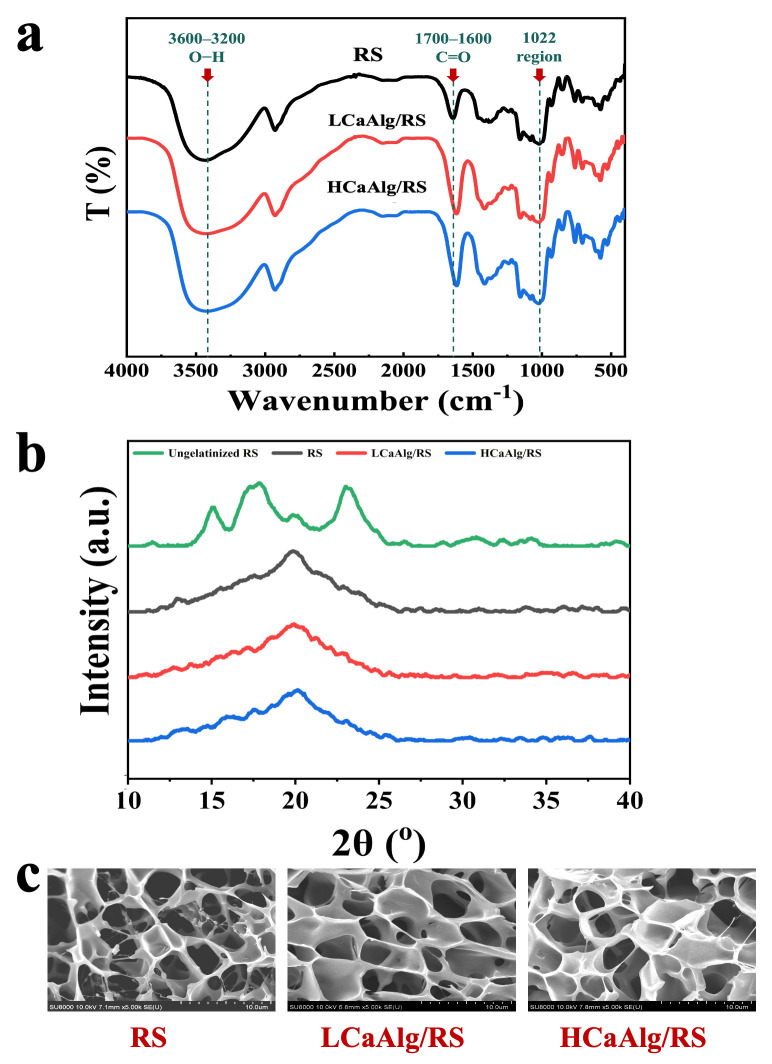
FT-IR spectra (**a**), XRD patterns (**b**), and Cryo-SEM images (×5000) (**c**) of Ca^2+^-crosslinked alginate/RS gel systems. In (**a**), the dashed lines indicate the main shifted bands, including O-H stretching, C=O stretching, and the band near 1022 cm^−1^ associated with the amorphous region and short-range molecular organization of starch.

FTIR, XRD, cryo-SEM were used to characterize the structural properties of rice starch gels, including intermolecular interactions and short-range molecular ordering [33,34], crystalline structure [35], and microstructural organization [36]. Figure 3a shows the FTIR spectra of RS and Ca^2+^-crosslinked alginate/RS samples. All samples exhibited similar characteristic absorption bands, indicating that no new functional groups were formed after Ca^2+^-induced alginate crosslinking. The O-H stretching band shifted from 3435.80 cm^−1^ in RS to 3422.50 cm^−1^ in LCaAlg/RS and 3428.84 cm^−1^ in HCaAlg/RS. The C=O stretching band shifted from 1640.81 cm^−1^ in RS to 1628.54 cm^−1^ and 1625.64 cm^−1^ in LCaAlg/RS and HCaAlg/RS, respectively. The band near 1022 cm^−1^, which is associated with the amorphous region and short-range molecular organization of starch [37], shifted from 1025.07 cm^−1^ in RS to 1022.50 cm^−1^ in LCaAlg/RS and 1028.14 cm^−1^ in HCaAlg/RS. Figure 3b shows the XRD patterns of each sample. The ungelatinized RS presents characteristic diffraction peaks at approximately 15°, 17°, and 23° (2θ), indicating a typical A-type crystalline structure. In contrast, all gelatinized samples exhibit broad and diffuse diffraction patterns, and the distinct crystalline peaks disappear. The diffraction profiles are mainly composed of a broad halo centered at ~20° (2θ), indicating a predominantly amorphous structure. After Ca^2+^-induced alginate crosslinking, no new diffraction peaks were observed, suggesting that no new crystalline phase was formed. Figure 3c shows the microstructure of each sample. RS presents a relatively loose and porous network structure, characterized by large and irregular pores and thin pore walls. In contrast, LCaAlg/RS and HCaAlg/RS appeared to exhibit a more continuous and compact network structure. With increasing alginate network content, HCaAlg/RS appeared to show a denser and more integrated structure, with thicker pore walls. The overall network was visually more continuous and uniformly distributed, with a lower degree of structural fragmentation.

These observations suggest that Ca^2+^-induced alginate crosslinking was associated with the formation of a more continuous gel network. Because no new absorption bands were observed in the FTIR spectra, the FTIR results do not indicate the formation of new functional groups or new covalent bonds. However, the shifts in the O-H and C=O bands suggest changes in the hydrogen-bonding environment and possible non-covalent interactions between alginate and starch chains. The shift in the 1022 cm^−1^ band further indicates changes in the local molecular environment of starch chains, especially in the amorphous region and short-range molecular organization [37]. This may be because Ca^2+^-crosslinked alginate interferes with the arrangement of starch chains, resulting in reduced structural ordering. This structural feature is consistent with the XRD results. No obvious crystalline diffraction peaks are observed in the samples after gelatinization, indicating that the system mainly remains in an amorphous state [38]. Although the rheological results showed increased viscosity and viscoelastic moduli in LCaAlg/RS and HCaAlg/RS, this does not necessarily indicate increased crystalline ordering. Rheological measurements mainly reflect the macroscopic strength and continuity of the gel network, whereas XRD detects long-range crystalline organization. Therefore, the strengthened gel behavior observed in the rheological analysis was mainly associated with Ca^2+^-induced alginate crosslinking, polymer interactions, and network formation rather than recrystallization. The slight differences in diffraction intensity and peak shape may be related to changes in the organization of the gel matrix. At the microstructural level, the Ca^2+^-crosslinked alginate/RS gel systems appeared to form a more continuous network structure, which may limit the rearrangement of starch chains and hinder the formation of ordered structures. These phenomena have also been reported in previous studies [39,40]. Overall, this constrained environment may also affect water distribution and molecular mobility in the gel system, providing a structural basis for changes in water behavior and digestive properties.

### 3.4. Ca^2+^-Crosslinked Alginate Network Reduces In Vitro Starch Digestibility in RS Gel Systems

**Figure 4:**
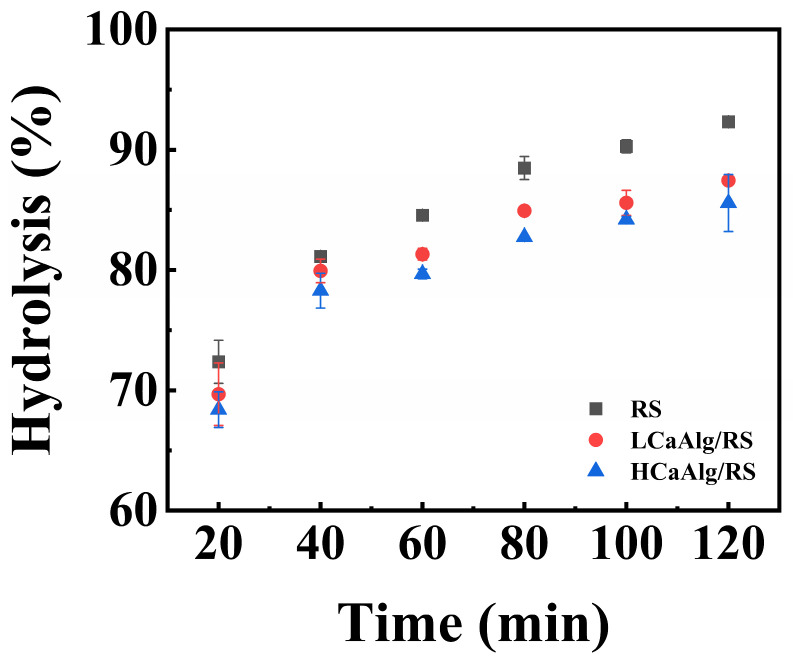
In vitro digestion degree of hydrolysis of Ca^2+^-crosslinked alginate/RS gel systems.

In terms of starch functionality, its digestive characteristics have attracted considerable attention. Excessive intake of foods rich in rapidly digestible starch may lead to unstable blood glucose levels. Over the long term, this may also reduce insulin sensitivity, which is not conducive to maintaining good health [41]. Figure 4 shows the effect of Ca^2+^-crosslinked alginate network formation on the in vitro enzymatic digestion characteristics of RS. All samples exhibit rapid digestion in the initial stage, followed by a slower hydrolysis phase. RS shows the highest degree of hydrolysis throughout the digestion process, reaching 92.3% at 120 min. In contrast, the hydrolysis rate of the Ca^2+^-crosslinked alginate/RS gel systems is reduced, with a more pronounced inhibitory effect observed in the HCaAlg/RS sample. After incubation for 120 min, the starch HRD of LCaAlg/RS and HCaAlg/RS are 87.5% and 85.6%, respectively. These results indicate that the Ca^2+^-crosslinked alginate/RS gel systems reduced the extent of enzymatic hydrolysis of RS under the present digestion conditions. Table 2 shows the contents of rapidly digestible starch (RDS), slowly digestible starch (SDS), resistant starch, and the kinetic parameters obtained from first-order kinetic fitting. Compared with RS, LCaAlg/RS and HCaAlg/RS showed a gradual decrease in RDS content and a corresponding increase in resistant starch content. The RDS content slightly decreased from 72.4% in RS to 69.7% in LCaAlg/RS and 68.4% in HCaAlg/RS, with a significant difference observed between RS and HCaAlg/RS (*p* < 0.05). The SDS content showed no significant difference among the three groups. In contrast, resistant starch increased from 7.7% in RS to 12.6% in LCaAlg/RS and 14.4% in HCaAlg/RS (*p* < 0.05). To further evaluate the digestion behavior, the hydrolysis curves were fitted using a first-order kinetic model. The equilibrium hydrolysis degree, C_∞_, decreased from 93.0% in RS to 88.0% and 86.0% in LCaAlg/RS and HCaAlg/RS, respectively. Similarly, the digestion rate constant, k, decreased from 0.027 min^−1^ in RS to 0.018 min^−1^ and 0.013 min^−1^ in LCaAlg/RS and HCaAlg/RS, respectively.

These results indicate that the main change in starch fractions was the increase in resistant starch, whereas the changes in RDS and SDS were relatively limited. SDS showed no significant difference among the three groups, and the decrease in RDS was mainly observed in HCaAlg/RS. Therefore, the reduced digestibility of the Ca^2+^-crosslinked alginate/RS gel systems was more clearly reflected by the lower HRD, decreased C_∞_ and k values, and increased resistant starch content. This result strengthens the digestion analysis by showing that the Ca^2+^-crosslinked alginate/RS gel systems affected not only endpoint hydrolysis, but also the overall hydrolysis process. The reduced digestibility may be associated with non-covalent interactions between alginate and starch molecules, as indicated by FTIR, and with the more continuous network structure observed by cryo-SEM, which may restrict enzyme diffusion and reduce enzyme accessibility to starch substrates, which is consistent with previous findings in hydrocolloid–starch systems [42]. In addition, the reduced free water mobility indicated by LF-NMR may further affect starch swelling and enzyme–substrate contact during digestion. Overall, the Ca^2+^-crosslinked alginate/RS gel systems showed reduced starch hydrolysis, lower rapidly digestible starch content, higher resistant starch content, and slower digestion kinetics, indicating enhanced digestion resistance of RS gels.

### 3.5. Ca^2+^-Crosslinked Alginate/RS Gel Systems Modulates Postprandial Glycemic Response in Mice

**Figure 5:**
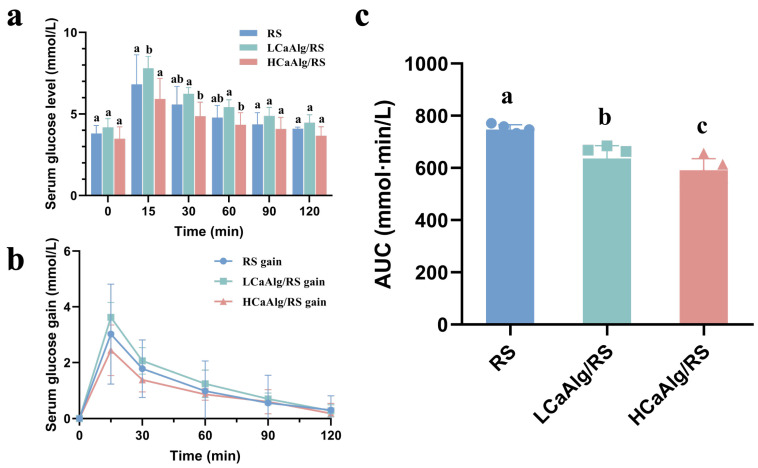
Postprandial blood glucose level (**a**), postprandial blood glucose response curves (**b**) and the corresponding incremental AUC (**c**) in mice after oral gavage of RS, LCaAlg/RS, and HCaAlg/RS. ^a–c^ Means with different letters in the same column are significantly different (*p* < 0.05).

Postprandial blood glucose response is widely used to evaluate the physiological effects of starch-based systems, as it reflects the dynamic release and absorption of glucose after ingestion [43]. Figure 5 shows the postprandial blood glucose level, response curves and the corresponding incremental area under the curve (AUC) after oral administration of RS, LCaAlg/RS, and HCaAlg/RS in mice. After ingestion, the average blood glucose levels in each group of mice show a trend of first increasing and then decreasing. Among them, the RS group exhibits the most rapid and pronounced increase in peak blood glucose. In contrast, the blood glucose levels of the LCaAlg/RS and HCaAlg/RS groups increase more slowly. The AUC values for the RS, LCaAlg/RS, and HCaAlg/RS groups are 747.2, 636.2, and 591.7 mmol·min/L, respectively. Compared with the RS group, the Ca^2+^-crosslinked alginate/RS gel systems were associated with lower postprandial blood glucose responses, with a more pronounced effect observed in the HCaAlg/RS group.

These results indicate that the Ca^2+^-crosslinked alginate/RS gel systems attenuated the postprandial glycemic response in mice. This effect may be partly associated with the slower release of digestible carbohydrates from the gel matrix, as supported by the lower HRD, reduced RDS content, increased resistant starch content, and decreased digestion rate constant observed in the in vitro digestion analysis. Previous studies have emphasized that the dynamic changes and fluctuations of glucose, rather than absolute blood glucose values, play a more important role in regulating feeding behavior [44]. In addition, several studies have shown that alginate intake can attenuate glycemic response. In various animal models, NaAlg has been reported to reduce intestinal glucose absorption [45]. Therefore, the lower AUC values observed in LCaAlg/RS and HCaAlg/RS may be related to the reduced starch hydrolysis and altered digestion behavior of the Ca^2+^-crosslinked alginate/RS gel systems.

### 3.6. Ca^2+^-Crosslinked Alginate/RS Gel Systems Slow Gastrointestinal Transit in Mice

**Figure 6:**
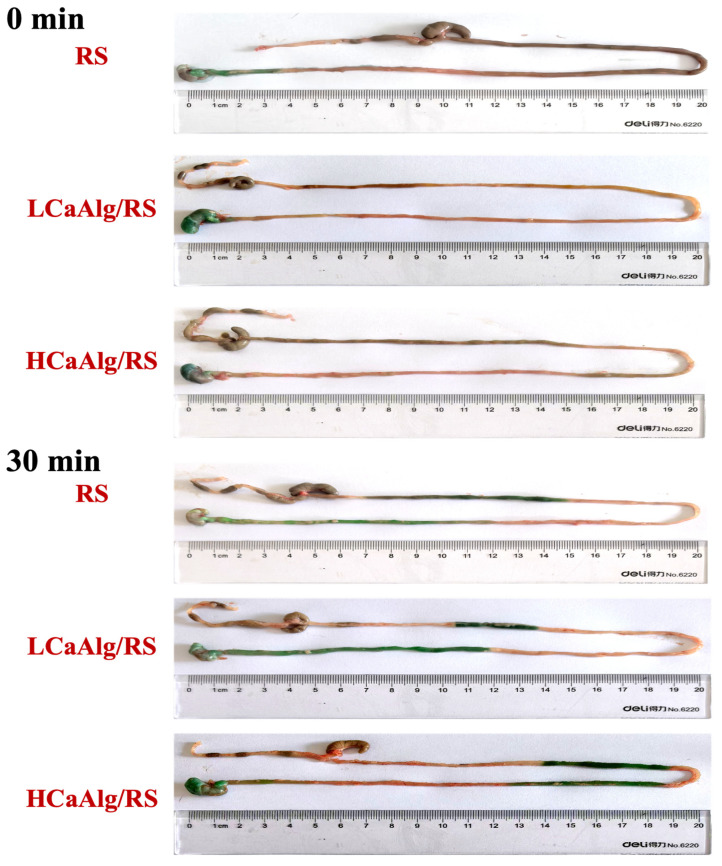
Gastrointestinal transit in mice following oral gavage of RS, LCaAlg/RS, and HCaAlg/RS containing a green ink marker. The blue-green appearance in the gastrointestinal tract corresponds to the administered green ink marker used to visualize the movement of gavaged contents. Representative images show the initial distribution of the marker at 0 min and its migration after 30 min.

In addition, this study employs the classical ink propulsion test to evaluate the impact of each sample on the gastrointestinal transit of mice. The green ink marker was used to visualize the movement of gavaged contents along the gastrointestinal tract, and the results are shown in Figure 6 and Table 3. Compared with the RS group, LCaAlg/RS and HCaAlg/RS slowed the migration of ink in the gastrointestinal tract. Specifically, at 0 min, the green ink marker mainly remained in the upper gastrointestinal tract in all groups, showing the initial distribution immediately after gavage. After 30 min, the RS group showed the longest migration distance of the ink marker, whereas LCaAlg/RS and HCaAlg/RS showed shorter migration distances and greater marker retention in the stomach and proximal intestine. The quantitative intestinal propulsion rate at 30 min is shown in Table 3. The intestinal propulsion rate decreased from 89.6% in the RS group to 82.2% in the LCaAlg/RS group and 75.3% in the HCaAlg/RS group. Different statistical letters indicate significant differences among the three groups (*p* < 0.05), confirming that the Ca^2+^-crosslinked alginate/RS gel systems significantly slowed intestinal propulsion.

These results suggest that the Ca^2+^-crosslinked alginate/RS gel systems were associated with slower gastrointestinal transit and delayed movement of gavaged contents along the digestive tract. This delay may affect the rate at which nutrients are delivered from the stomach to the small intestine, thereby reducing the rate of glucose absorption. This effect is consistent with the attenuated postprandial blood glucose response observed in LCaAlg/RS and HCaAlg/RS. Previous studies have also reported that alginate can increase gastrointestinal viscosity and delay gastric emptying, thereby slowing nutrient release and absorption [46]. Overall, these findings indicate that the Ca^2+^-crosslinked alginate/RS gel systems can regulate postprandial blood glucose response not only by modulating starch digestibility, but also by altering gastrointestinal dynamics, highlighting its role in controlling nutrient delivery and absorption.

## 4. Conclusions

In this study, NaAlg was incorporated into an RS gel system and subsequently crosslinked with Ca^2+^ to form the CaAlg network, and the effects of the resulting Ca^2+^-crosslinked alginate/RS gel systems on the physicochemical properties, digestion behavior, and physiological response of RS. The results show that Ca^2+^-crosslinked alginate network formation increased the viscosity and viscoelastic moduli of RS gels and was associated with the formation of a more continuous gel network. Structural characterization by FTIR, XRD, and cryo-SEM further confirms that the Ca^2+^-crosslinked alginate/RS gel systems showed enhanced intermolecular interactions, while all gelatinized samples mainly remained in an amorphous state without the formation of new crystalline structures. These structural and physicochemical changes are closely related to the digestion behavior of RS. Compared with RS, the Ca^2+^-crosslinked alginate/RS gel systems showed lower starch hydrolysis, reduced RDS content, increased resistant starch content, and slower digestion kinetics, as reflected by the decreased C_∞_ and k values. In vivo experiments further showed that LCaAlg/RS and HCaAlg/RS were associated with lower postprandial glycemic responses and slower gastrointestinal transit in mice. These findings suggest that Ca^2+^-crosslinked alginate network formation may modulate the functional behavior of RS gels through combined effects on gel structure, water distribution, starch digestion, and gastrointestinal transit.

Nevertheless, NaAlg-only and CaCl_2_-only controls were not included, which limited the ability to distinguish the individual contributions of alginate viscosity, calcium ions, network formation, and starch dilution. Therefore, the observed effects should be interpreted as the combined effects of the formulated Ca^2+^-crosslinked alginate/RS gel systems. Overall, this study provides a useful basis for developing RS-based gel systems with improved digestion resistance and potential glycemic control properties.

## Figures and Tables

**Table 1:** Relaxation time and peak area of water distribution in Ca^2+^-crosslinked alginate/RS gel systems.

Samples	T_23_ (ms)	A_23_
RS	265.6 ± 0.0 ^b^	1934.8 ± 90.7 ^c^
LCaAlg/RS	231.0 ± 0.0 ^a^	1839.0 ± 57.3 ^b^
HCaAlg/RS	231.0 ± 0.0 ^a^	1770.9 ± 77.4 ^a^

All data represent the mean of triplicates. ^a–^^c^ Means with different letters in the same column are significantly different (*p* < 0.05).

**Table 2:** Digestive characteristics and first-order kinetic parameters of Ca^2+^-crosslinked alginate/RS gel systems.

Samples	RDS (%)	SDS (%)	Resistant Starch (%)	C_∞_ (%)	k (min^−1^)
RS	72.4 ± 1.8 ^b^	20.0 ± 1.4 ^a^	7.7 ± 0.4 ^a^	93.0 ± 0.4 ^b^	0.027 ± 0.001 ^b^
LCaAlg/RS	69.7 ± 2.6 ^b^	17.8 ± 2.8 ^a^	12.6 ± 0.4 ^b^	88.0 ± 0.5 ^a^	0.018 ± 0.003 ^a^
HCaAlg/RS	68.4 ± 1.5 ^a^	17.2 ± 3.8 ^a^	14.4 ± 2.4 ^c^	86.0 ± 2.0 ^a^	0.013 ± 0.006 ^a^

All data represent the mean of triplicates. ^a–^^c^ Means with different letters in the same column are significantly different (*p* < 0.05).

**Table 3:** Intestinal propulsion rate of Ca^2+^-crosslinked alginate/RS gel systems.

Samples	Intestinal Propulsion Rate (%)
RS	89.6 ± 0.8 ^a^
LCaAlg/RS	82.2 ± 2.3 ^b^
HCaAlg/RS	75.3 ± 2.6 ^c^

All data are expressed as mean ± SD (*n* = 5). ^a–^^c^ Means with different letters in the same column are significantly different (*p* < 0.05).

## Data Availability

The original contributions presented in this study are included in the article/Supplementary Materials. Further inquiries can be directed to the corresponding author.

## Supplementary Materials

The following supporting information can be downloaded at: https://www.mdpi.com/article/10.3390/foods15122146/s1, Table S1. Water separation rate of Ca^2+^-crosslinked alginate/RS gel systems after freeze–thaw treatment. Figure S1. Schematic diagram of the animal experimental design.

## Abbreviation

RS; Rice Starch; NaAlg; Sodium alginate; CaAlg; Calcium alginate; CaCl_2_; Calcium chloride; LF-NMR; Low-field nuclear magnetic resonance; XRD; X-ray diffraction; Cryo-SEM; Cold-field scanning electron microscopy; FT-IR; Fourier-transform infrared spectroscopy; RDS; Rapidly digestible starch; SDS; Slowly digestible starch; AUC; Area under the curve; HRD; Hydrolysis rate of digestion
