# Supplementary material for: Ca2+-Crosslinked Alginate Network Attenuates Starch Digestibility and Postprandial Glycemic Response in Rice Starch Gels
Source: Foods. 2026 Jun 14;15(12):2146. doi: 10.3390/foods15122146 (PMC13297811; doi:10.3390/foods15122146)

Supplementary Material

Table caption:

Table S1. Water separation rate of Ca^{2+}-crosslinked alginate/RS gel systems after freeze–thaw treatment.

**Samples**	Water separation rate **(%)**
RS	40.3 ± 0.5^{c}
LCaAlg/RS	38.4 ± 1.0^{b}
HCaAlg/RS	35.3 ± 0.6^{a}

All data represent the mean of triplicates. Different letters in the same column indicate significant differences ($p < 0.05$).

Figures caption:

Figure S1. Schematic diagram of the animal experimental design.

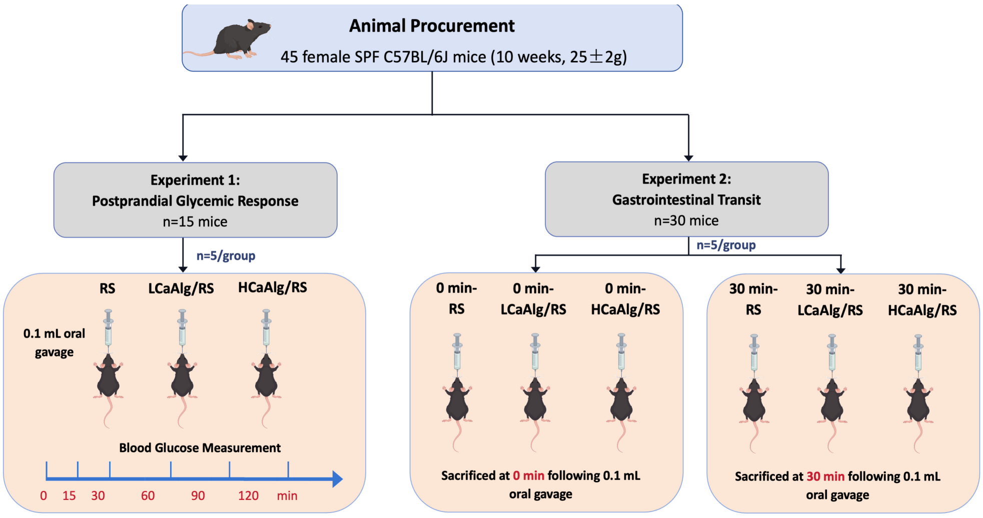

Supplement: Supplementary file 1 [file foods-15-02146-s001.zip › foods-4319091-supplementary.pdf]
